# Oxidative Stress in Animal Models of Acute and Chronic Renal Failure

**DOI:** 10.1155/2019/8690805

**Published:** 2019-02-11

**Authors:** Marianna Gyurászová, Alexandra Gaál Kovalčíková, Emese Renczés, Katarína Kmeťová, Peter Celec, Janka Bábíčková, Ľubomíra Tóthová

**Affiliations:** ^1^Institute of Molecular Biomedicine, Faculty of Medicine, Comenius University, Bratislava, Slovakia; ^2^Department of Pediatrics, National Institute of Children's Diseases and Faculty of Medicine, Comenius University, Bratislava, Slovakia; ^3^Institute of Pathophysiology, Faculty of Medicine, Comenius University, Bratislava, Slovakia; ^4^Department of Molecular Biology, Faculty of Natural Sciences, Comenius University, Bratislava, Slovakia; ^5^Department of Clinical Medicine, University of Bergen, Bergen, Norway

## Abstract

**Introduction:**

Kidney disease is a worldwide health and economic burden, with rising prevalence. The search for biomarkers for earlier and more effective disease screening and monitoring is needed. Oxidative stress has been linked to both, acute kidney injury (AKI) and chronic kidney disease (CKD). The aim of our study was to investigate whether the concentrations of systemic markers of oxidative stress and antioxidant status are affected by AKI and CKD, and to identify potential biomarkers.

**Methods:**

In adult male Wistar rats, AKI was induced by bilateral nephrectomy, and CKD was induced by 5/6 nephrectomy. Blood was collected 48 hours after surgery in AKI and 6 months after surgery in CKD. Advanced oxidation protein products (AOPP), thiobarbituric acid reactive substances (TBARS), advanced glycation end products (AGEs), fructosamine, total antioxidant capacity (TAC), and ferric reducing antioxidant power (FRAP) were measured.

**Results:**

Impaired renal function was confirmed by high concentrations of plasma creatinine and urea in AKI and CKD animals. AOPP and fructosamine were higher by 100% and 54% in AKI, respectively, and by 100% and 199% in CKD, respectively, when compared to corresponding control groups. Similarly, there was approximately a twofold increase in AGEs (by 92%) and TAC (by 102%) during AKI. In CKD, concentrations of FRAP, as an antioxidative status marker, were doubled (by 107%) when compared to the control group, but concentration of TAC, another marker of antioxidative status, did not differ between the groups.

**Conclusions:**

AKI and CKD led to increased systemic oxidative stress. AOPP and fructosamine could be considered potential biomarkers for both, acute and chronic kidney damage. On the other hand, AGEs, TAC, and FRAP seem to be disease specific, which could help to differentiate between acute and chronic kidney injuries. However, this needs further validation in clinical studies.

## 1. Introduction

Kidney disease can be classified into two types: acute kidney injury (AKI) and chronic kidney disease (CKD). The global prevalence of both forms of kidney disease is rising continuously, in part due to the aging population and in part due to a global increase in the prevalence of hypertension and diabetes. AKI is characterized by a sudden loss of kidney function within 7 days. CKD develops due to a structural or functional kidney irregularity that persists for at least 3 months. Both AKI and CKD result in the accumulation of toxic end products of nitrogen metabolism and creatinine in the blood [1–3]. Research is focused on the search for more effective and less costly therapies of AKI and end-stage CKD. More importantly, the search for screening methods of earlier detection and more effective disease monitoring is ongoing [4].

Oxidative stress is a state of imbalance between the generation of prooxidants and the number of antioxidants present in favor of prooxidants. Free radicals and nonradical oxidants alter biomolecules, mainly lipids, proteins, and nucleic acids, ultimately leading to cell death. Mitochondria provide the main energy source for cells. In the kidney, renal tubular cells are especially rich in mitochondria, because the reabsorption of solutes is highly energy demanding. Renal tubules are particularly vulnerable to oxidative stress and damage, since mitochondria are one of the main sites of intracellular free radical production via the respiratory chain and NADPH oxidases [5–7]. Increased production of prooxidants is involved in many pathological pathways of AKI and CKD [8].

In CKD, impaired mitochondrial function and enhanced mitochondrial reactive oxygen species (ROS) has been proposed as one of the causes of elevated oxidative stress [9]. Increased production of mitochondrial ROS was shown in the kidneys of diabetic mice [10–12]. In AKI, oxidative damage and decreased antioxidant status can develop in the renal tissue due to ischemia and toxic damage [13–15]. Also, oxidative stress is a key factor in the pathogenesis of rhabdomyolysis-induced myoglobinuric AKI [16]. Another source of oxidative stress in later stages of CKD may be the presence of uremic toxins and dysregulated metabolic waste disposal [17–23]. In patients with CKD, uremia-specific risk factors, including volume expansion, chronic inflammation, anemia, or a microinflammatory state, are associated with systemic oxidative stress that in turn could cause inflammation and further tissue damage [6]. Thus, oxidative stress can affect the progression of renal disease as well in a bidirectional manner. It can aggravate inflammation, contribute to the development of fibrosis, via enhanced inflammation and trigger signaling pathways leading to renal tubular cell death. Fibrosis and inflammation might increase further formation of ROS [24]. According to the above-mentioned studies, it has been shown that oxidative stress is strongly associated with AKI and CKD and their complications. However, it is still questionable whether an elevated production of prooxidants is the only reason of oxidative stress. Uremic toxins bolster inflammation along with oxidative stress by priming polymorphonuclear leukocytes and triggering an innate immune response. Uric acid synthesis can further promote oxidative stress through the activity of xanthine oxidoreductase. On the other hand, the uric acid itself can act as an antioxidant [25–27].

Thus, oxidative stress and antioxidant status markers need to be analyzed in detail. Oxidative status is commonly assessed in the plasma by measuring the concentrations of damaged biomolecules by free radicals and other oxidants. Lipid peroxidation is routinely measured by the thiobarbituric acid-reacting substance (TBARS) method [28]. For the evaluation of protein oxidation, advanced oxidation protein products (AOPP) are assessed [29]. Advanced glycation end products (AGEs) along with fructosamine are formed as late-stage products of nonenzymatic glycation of amino groups of proteins by the carbonyl compound of sugars, and they are used as markers of carbonyl stress [30]. Total antioxidant capacity (TAC) and ferric reducing antioxidant power (FRAP) are commonly used methods for the assessment of the antioxidant status [31, 32].

To our knowledge, only very few studies describing oxidative stress and antioxidant status markers in detail in both AKI and CKD have been published. The most recent one suggests that salivary AOPP could be a suitable marker for the detection of CKD in children with 92% sensitivity and specificity [33]. Since AOPP are nonspecific, a panel of oxidative stress markers would be more helpful in the detection of CKD or AKI. Therefore, the main goal of this study was to experimentally determine the effect of acute kidney injury and chronic kidney disease on the systemic oxidative status. In addition, AKI and CKD were analyzed in parallel to search for possible differences between the two conditions, which might prove useful in the differentiation between progressive CKD and AKI as a complication of CKD or alone.

## 2. Methods

### 2.1. Animals

Twelve-week-old adult male Wistar rats were used in this experiment (*n* = 40 in total, weighing 294 ± 79 grams, Anlab, Prague, Czech Republic). Rats were housed in standard cages with wood chip bedding, in a room with an ambient temperature of 22 ± 1°C, 40-50% humidity, and a 12/12-hour light/dark cycle. All rats had *ad libitum* access to standard rodent chow and tap water throughout the experiment. This study was approved by the Ethics Committee of the Institute of Pathophysiology, Comenius University, and was carried out according to relevant national legislation.

### 2.2. Modeling of Acute Kidney Injury

Bilateral nephrectomy was performed to model AKI. Rats were divided into two groups, a bilateral nephrectomy group (BNex, *n* = 11) and a sham group (BNex sham, *n* = 6). The animals were bilaterally nephrectomised in one surgical session, as described previously [34]. Animals were anesthetized by ketamine (100 mg/kg, Richter Pharma AG, Wels, Austria) and xylazine (10 mg/kg, Ecuphar N.V., Oostkamp, Belgium) administered by intraperitoneal injection. A midline incision was made in the BNex group; the kidneys were exposed and decapsulated. Renal pedicles were tied off with a suture, and the kidneys were removed. The incision was closed with an absorbable suture. Animals in the sham group underwent sham surgery. Both kidneys of the rats in this group were decapsulated. Animals were sacrificed 48 hours after surgery, under general anesthesia. Blood was collected into EDTA-coated blood collection tubes (Sarstedt, Numbrecht, Germany) from the abdominal aorta. Plasma was obtained by centrifugation (5000 g for 5 minutes) and stored at -20°C until further analysis.

### 2.3. Modeling of Chronic Kidney Disease

To model CKD, 5/6 nephrectomy was conducted. Rats were divided into two groups, a 5/6 nephrectomised (5/6 Nex, *n* = 14) and a sham group (5/6 Nex sham, *n* = 9). The animals in the 5/6 Nex group underwent subtotal (5/6) nephrectomy in two surgical steps, as reported previously [35]. Briefly, animals were anesthetized by ketamine (100 mg/kg, Richter Pharma AG, Wels, Austria) and xylazine (10 mg/kg, Ecuphar N.V., Oostkamp, Belgium) administered by intraperitoneal injection. A midline incision was made on the left side, and the left kidney was exposed. The kidney was decapsulated, and the upper and lower kidney poles were removed. Bleeding was stopped by Gelaspon (Chauvin Ankerpharm GmbH, Rudolstadt, Germany), and the incision was closed with an absorbable suture. After the recovery period (14 days later), a similar incision was made on the right side, the renal vessels were ligated, and the decapsulated kidney was removed. Animals in the 5/6 Nex sham group underwent sham surgery. The kidneys of the rats in this group were decapsulated at the time of the first surgery. Animals were sacrificed 6 months after surgery under general anesthesia. Animals were placed in metabolic cages (4 hours) for urine collection. Plasma samples were obtained in the same manner as described in the AKI model.

### 2.4. Biochemical Analysis

Three hundred and fifty microliters of plasma were used to measure plasma creatinine, urea, and albuminuria concentrations using the Biolis 24i Premium automated clinical analyzer (Tokyo Boeki Medical System Ltd., Tokyo, Japan) [36]. The principle of urea measurement is based on the hydrolysis of urea by urease to form ammonium and carbonate. The second step is based on the reaction 2-oxoglutarate that reacts with ammonium in the presence of glutamate dehydrogenase (GLDH) and the coenzyme NADH. This reaction produces L-glutamate [37]. The measurement of creatinine is based on its reaction with picric acid in alkaline conditions that results in a reddish complex [38]. The plasma concentrations of kidney injury molecule 1 (KIM-1) were measured using the commercial rat KIM-1 ELISA kit (R&D Systems Inc., Abingdon, UK) according to the manufacturer's protocol.

### 2.5. Oxidative Stress and Antioxidant Status Analysis

Markers of oxidative stress and antioxidant status were measured in plasma samples using a Synergy H1 multimode microplate reader (BioTek Instruments, Inc., Winooski, VT, USA).

TBARS were measured by pipetting 20 *μ*l of samples or standards (1,1,3,3-tetraethoxypropane), 30 *μ*l of distilled water, 20 *μ*l of 0.67% thiobarbituric acid, and 20 *μ*l of glacial acetic acid in a microtiter plate. The plates were mixed and incubated for 45 minutes at 95°C. 100 *μ*l of n-butanol was added into the samples, and the plates were centrifuged (2000 g/10 min/4°C). Seventy microliters of the upper phase was transferred into a new microtiter plate, and fluorescence was measured at _ex_ = 515 nm and *λ*_em_ = 535 nm. AOPP was assessed by mixing 200 *μ*l of samples and standards (chloramine T mixed with potassium iodide) with 20 *μ*l of glacial acetic acid for 2 minutes. Absorbance was measured at 340 nm. AGEs were measured by pipetting 20 *μ*l of sample or standards (AGE-BSA) and 180 *μ*l of PBS into a dark microtiter plate, vortexing and measuring fluorescence at _ex_ = 370 nm and *λ*_em_ = 440 nm. For fructosamine measurement, 20 *μ*l of the samples and standards (16 mmol/l 1-deoxy-morpholino-D-fructose) was mixed with 100 *μ*l of 0.25 mmol/l nitroblue tetrazolium containing 1 mmol/l nitroblue tetrazolium and 0.1 mol/l sodium carbonate buffer (pH = 10.35). Samples were incubated at 37°C for 15 minutes. Absorbance was measured at 530 nm.

For TAC measurement, samples were mixed with acetate buffer (pH = 5.8). Absorbance was measured at 660 nm as blank. When the ABTS solution (2.2′-azino-bis(3-ethylbenzthiazoline-6-sulphonic acid with acetate buffer)) was added, the absorbance was measured again at 660 nm. The blank absorbance values were subtracted from the values obtained by the second measurement. For FRAP assessment, 200 *μ*l of warmed (37°C) FRAP reagent (containing acetate buffer (pH = 3.6), tripyridyl-s-triazine, FeCl_3_∗6H_2_O, and water) was pipetted into a microtiter plate, and absorbance was measured as blank. Afterwards, 20 *μ*l of samples and standards (100 mmol/l FeSO_4_∗7H_2_O) was added. Absorbance was measured again at 530 nm. The blank absorbance values were subtracted from the values obtained by the second measurement [39].

For the measurement of proteins, 10 *μ*l of the samples and standards (bovine serum albumin) was mixed with 200 *μ*l of the working solution (bicinchoninic acid and copper sulphate, 49 : 1 ratio, respectively). The plate was incubated at 37°C for 30 minutes. After cooling, absorbance was measured at 562 nm. All markers measured in plasma were normalized to plasma protein concentrations. Markers measured in urine were normalized to urinary creatinine concentrations.

### 2.6. Statistical Analysis

GraphPad Prism 5.0 (GraphPad Software, San Diego, CA, USA) was used for the statistical analyses. After testing for normality with the D'Agostino-Pearson omnibus test, data were analyzed with the Mann–Whitney *U* test. The Spearman correlation test and linear regression were used to evaluate the linear associations between quantitative variables. A value of *p* < 0.05 was considered statistically significant.

## 3. Results

**Figure 1:**
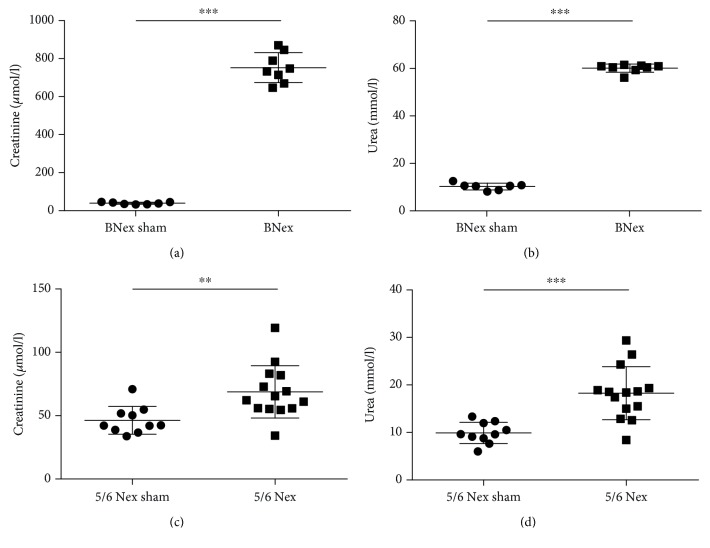
Renal function parameters in rats with acute and chronic kidney injury and sham-operated rats. (a) Plasma creatinine in bilaterally nephrectomised (BNex) and sham (BNex sham) rats, (b) plasma urea in BNex and BNex sham rats, (c) plasma creatinine in 5/6 nephrectomised (5/6 Nex) and sham (5/6 Nex sham) rats, and (d) plasma urea in 5/6 Nex and 5/6 Nex sham rats. ^∗∗^*p* < 0.01, ^∗∗∗^*p* < 0.001.

Concentrations of creatinine in the plasma of the BNex group were 20-fold higher when compared to the BNex sham group (Figure 1(a); *U* = 0, *p* < 0.001). Blood urea in the BNex group was significantly higher when compared to the BNex sham group by 484% (Figure 1(b); *U* = 0, *p* < 0.001). Plasma creatinine concentrations in the 5/6 Nex group were significantly higher than in the 5/6 Nex sham group by 48% (Figure 1(c); *U* = 18, *p* < 0.01). Blood urea was 2-fold higher in the 5/6 Nex group than in the 5/6 Nex sham group (Figure 1(d); *U* = 10, *p* < 0.001). One animal in the BNex group did not show elevated levels of plasma creatinine (37.56 *μ*mol/l), nor urea (11.70 mmol/l), which would comply with the modeling of AKI and was thus removed from further analyses.

**Figure 2:**
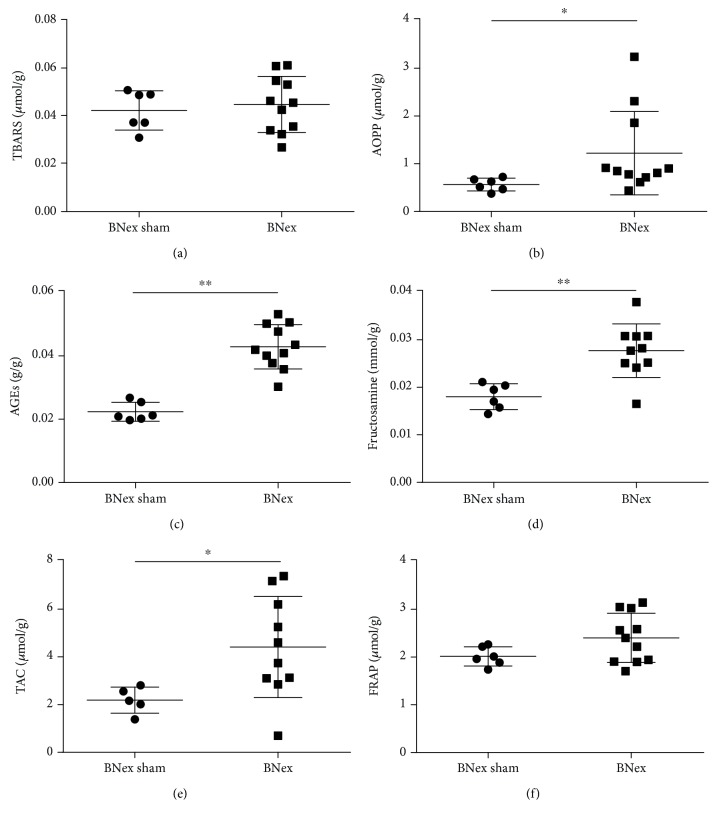
Oxidative stress and antioxidant status markers in the plasma of bilaterally nephrectomised (BNex) and sham (BNex sham) rats. (a) TBARS: thiobarbituric acid reactive substances, (b) AOPP: advanced oxidation protein products, (c) AGEs: advanced glycation end products, (d) fructosamine, (e) TAC: total antioxidant capacity, and (f) FRAP: ferric reducing antioxidant power. ^∗^*p* < 0.05, ^∗∗^*p* < 0.01.

**Figure 3:**
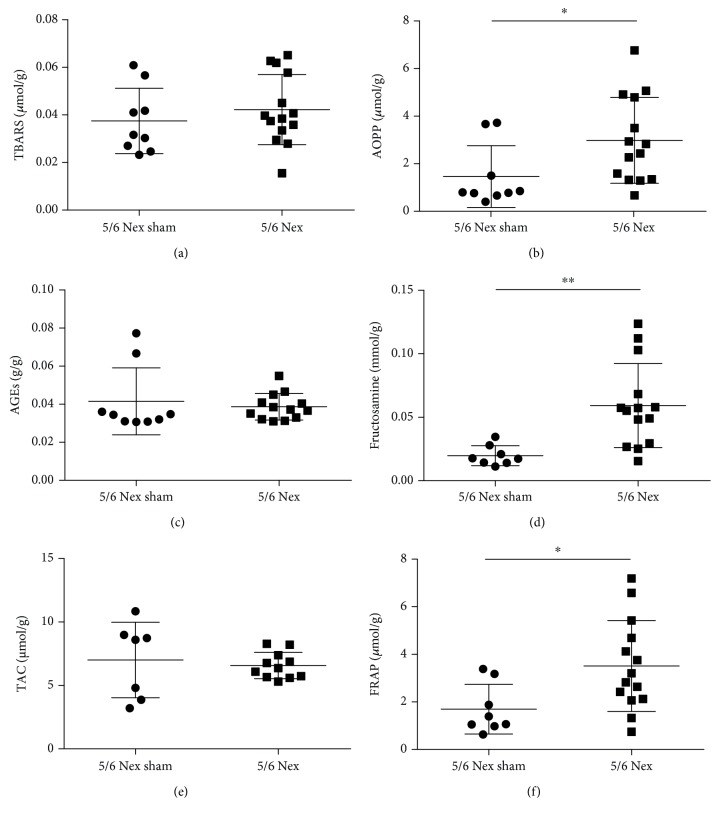
Oxidative stress and antioxidant status markers in the plasma of 5/6 nephrectomised (5/6 Nex) and sham (5/6 Nex sham) rats. (a) TBARS: thiobarbituric acid reactive substances, (b) AOPP: advanced oxidation protein products, (c) AGEs: advanced glycation end products, (d) fructosamine, (e) TAC: total antioxidant capacity, and (f) FRAP: ferric reducing antioxidant power. ^∗^*p* < 0.05, ^∗∗^*p* < 0.01.

There were no differences between the BNex and the BNex sham groups in TBARS concentrations (Figure 2(a)). In plasma TBARS, no significant differences were found between the 5/6 Nex and the 5/6 Nex sham groups either (Figure 3(a)). Plasma AOPP concentrations were 2-fold higher in the BNex group, when compared to the BNex sham group (Figure 2(b); *U* = 9, *p* < 0.05). In plasma AOPP, a significant difference was found between the 5/6 Nex and the 5/6 Nex sham groups, the first being higher by 102% (Figure 3(b); *U* = 28, *p* < 0.05).

AGE concentrations were 2-fold higher in the plasma of the BNex group, when compared to the BNex sham group (Figure 2(c); *U* = 0, *p* < 0.01). No significant differences were found in plasma AGE concentration between the 5/6 Nex and the 5/6 Nex sham groups (Figure 3(c)). Fructosamine concentrations differed significantly between the BNex and the BNex sham group as well, the BNex group being higher by 54% (Figure 2(d); *U* = 4, *p* < 0.01). In plasma fructosamine, a significant difference was found between the 5/6 Nex and the 5/6 Nex sham groups. The 5/6 Nex group had 3-fold higher fructosamine concentrations (Figure 3(d); *U* = 10, *p* < 0.01). Between the BNex and the BNex sham groups, there was a significant difference in TAC, the BNex being 2-fold higher (Figure 2(e); *U* = 5, *p* < 0.05). TAC concentrations in plasma did not differ between the 5/6 Nex and the 5/6 Nex sham groups (Figure 3(e)). There were no differences between the BNex and the BNex sham groups in FRAP concentrations (Figure 2(f)). In plasma FRAP, a significant difference was found between the 5/6 Nex and the 5/6 Nex sham groups, the first being 2-fold higher (Figure 3(f); *U* = 22, *p* < 0.05).

**Figure 4:**
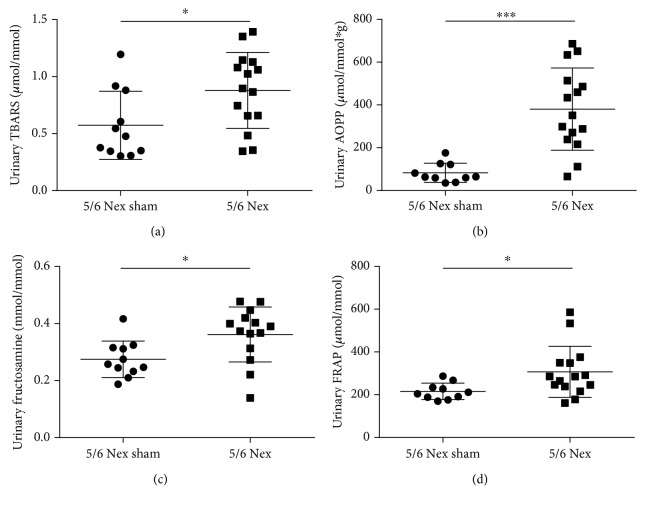
Oxidative stress and antioxidant status markers in the urine of 5/6 nephrectomised (5/6 Nex) and sham (5/6 Nex sham) rats. (a) TBARS: thiobarbituric acid reactive substances, (b) AOPP: advanced oxidation protein products, (c) fructosamine, and (d) FRAP: ferric reducing antioxidant power. ^∗^*p* < 0.05, ^∗∗∗^*p* < 0.001.

Additionally, to the plasma concentrations, TBARS, AOPP, fructosamine, and FRAP were all measurable in the urine of 5/6 Nex rats. Of these, AOPP differed most significantly from the sham group. AOPP in 5/6 Nex were 2-fold higher in urine when compared to 5/6 Nex sham group (Figure 4(b); *U* = 7, *p* < 0.001). TBARS, fructosamine, and FRAP in urine were increased in the 5/6 Nex group by approximately 50% (Figures 4(a), 4(c), and 4(d); *U* = 40, 34, and 32, respectively, *p* < 0.05 for all three markers) when compared to the control group.

In 5/6 Nex as a model of CKD, urinary AOPP significantly and positively correlated with urinary KIM-1 (*r* = 0.70; *p* < 0.05). Also, urinary fructosamine and FRAP significantly correlated with ACR (*r* = 0.72 and *r* = 0.82; *p* < 0.05, respectively) and albuminuria (*r* = 0.62 and *r* = 0.82; *p* < 0.05, respectively), but neither with plasma nor with urinary KIM-1 (Table 1).

## 4. Discussion

The results of this experiment confirmed the association between elevated systemic oxidative stress and acute and chronic renal failure. Although proteins are relatively resistant to damage by prooxidants, AOPP concentrations were 2-fold higher in rats with both acute and chronic renal failure compared to their sham-operated counterparts. A 2-fold increase in AOPP was present not only in plasma but also in the urine of CKD rats. These findings are in line with previous studies in AKI and CKD patients [40, 41]. It has been proposed that AOPP not only are associated with CKD but also have a pathogenic role in CKD progression through cellular and molecular mechanisms. These mechanisms include triggering a cascade of signaling events that lead to superoxide generation, NF-*κ*B activation, the overproduction of extracellular matrix, apoptosis of podocytes, endothelial inflammation, and monocyte activation [42, 43]. Moreover, elevated AOPP were found to be associated with poor prognosis of patients with IgA nephropathy [44]. On the other hand, during CKD, the excessive glomerular protein leakage might contribute to the absolute numbers of AOPP.

This study found no differences between 5/6 Nex or BNex animals and the sham groups in oxidized lipid concentrations measured by TBARS in plasma, contrary to other studies. A previous study showed that products of lipid peroxidation could be increasing in AKI in a time-dependent manner. It could be argued that these damaged lipids would have increased should the experiment lasted longer, although, in the mentioned studies, lipid peroxidation products were already significantly higher after 48 hours in rats with AKI [45, 46]. On the other hand, urinary TBARS were higher in CKD rats, suggesting their clearance from circulation despite reduced renal function. Unfortunately, due to the anuria of the BNex rats, we were unable to determine the urinary levels of TBARS in the acute setting.

Products of carbonyl stress, such as fructosamine and AGEs, are produced during aging and accumulate in circulation and tissues. Kidneys play an important part in their disposal, and fructosamine and AGEs have been shown to be elevated when kidney function is compromised [47, 48]. The results of this experiment showed that the concentration of fructosamine was higher in AKI and CKD, but AGEs were higher only in the AKI model. This could indicate that the remaining kidney function in the mild 5/6 Nex model of CKD was sufficient to filter AGEs from circulation. The reasons of the accumulation of AGEs during uremia are not fully understood. Increased concentration of circulating AGEs in patients with kidney diseases may result from an increased production due to uremia-related consequences or decreased renal disposal [49]. According to previous results, low molecular weight AGEs are easily removed from the body by renal clearance [50]. Thus, it seems that a partial maintenance of low molecular weight AGE disposal by the damaged kidneys prevented the elevation of plasma AGE concentrations in 5/6 Nex. However, it has also been shown that one fraction of plasma AGEs is linked to binding proteins, mainly albumin in uremic patients. Glomerular filtration or dialysis does not remove these proteins. Thus, accumulation of higher molecular weight AGEs could not be explained by decreased renal clearance [51]. In addition, the elevated urinary levels of fructosamine in 5/6 Nex rats confirm their clearance via the kidneys.

The results also showed that the concentrations of antioxidant status markers were higher in the 5/6 Nex and BNex groups than in controls—more precisely, FRAP was higher in the 5/6 Nex group, and TAC was found to be elevated in the BNex group when compared to sham animals. These results are in line with a different study showing higher TAC in a rat model of AKI than in sham-operated animals [52]. Another study involving CKD patients has shown higher FRAP concentrations in patients than in controls [53]. In addition, urinary FRAP levels were also elevated in 5/6 Nex, confirming the situation observed in plasma. Although we cannot fully explain the difference between AKI and CKD, this could be potentially used as a differentiating marker between AKI and CKD. It is assumed that the antioxidant activity rose as an answer to present systemic oxidative stress in both disease states. Paradoxically, the antioxidant status may increase in renal disease because of the accumulation of uremic toxins with scavenging capacity, such as indole derivatives and hippurate [48, 52]. In addition, the difference might be the result of the differences of the measured substances between the two methods. While TAC includes the SH/thiol groups present in proteins and reduced glutathione, these are not detected by the FRAP method. Due to the complete anuria in the BNex rats compared to the known proteinuria in the 5/6 Nex model, the higher TAC in BNex might be attributed to a higher amount of small molecular weight proteins in the plasma of BNex rats [54].

To further address the applicability of the oxidative stress markers in renal disease, we correlated them with the standard plasmatic and urinary markers of renal function and injury.

In CKD, we found a negative correlation between plasma AGEs vs. plasma creatinine and uKIM-1. Whether this observation is of any prognostic or diagnostic value is currently unknown. In the urine, there was a positive correlation between AOPP and uKIM-1, suggesting that urinary AOPP might closely reflect proximal tubular injury. On the other hand, urinary fructosamine and FRAP correlated with ACR and albuminuria, but not with uKIM-1, suggesting that they might more closely reflect glomerular injury. However, further studies are needed to prove these assumptions.

The limitation of this study is that the urinary concentration of oxidative stress and antioxidant status markers could not be assessed in BNex rats, due to anuria which is typical for this model [55]. Further studies will be aimed at the models with residual kidney function, which are not completely anuric.

In conclusion, this study confirms the association of AKI and CKD with elevated systemic oxidative stress markers. Furthermore, to our knowledge, this study is the first to describe this particular set of oxidative stress and antioxidant status markers measured parallel in AKI and CKD. It shows that oxidative damage to proteins is apparent in both disease states in plasma and in urine. Nevertheless, we do not report elevated lipid peroxidation in kidney disease in plasma, neither in AKI nor in CKD. Interestingly, in the urine, we found elevated markers of lipid peroxidation in CKD, which might suggest urine as a superior fluid for the determination of lipid peroxidation in CKD. Carbonyl stress is apparent by an increase in fructosamine in both disease states, but the remnant kidney function in CKD appears to be sufficient to excrete AGEs of lower molecular weight. Antioxidant status can also increase in AKI and CKD, likely due to an increase in uremic toxins with antioxidant capacity. However different antioxidant systems probably take part in increased oxidative stress during CKD and AKI. Future studies should be focused on AOPP and fructosamine as potential biomarkers, and data on their dynamics and urinary concentrations in AKI should be gathered. Further clarification of different antioxidant mechanisms during AKI and CKD is needed.

## Figures and Tables

**Table 1:** Correlation coefficients (Spearman) between plasma markers, thiobarbituric acid reactive substances (TBARS), advanced oxidation protein products (AOPP), advanced glycation end products (AGEs), fructosamine, total antioxidant capacity (TAC), and ferric reducing antioxidant power (FRAP), and urinary markers, TBARS, AOPP, fructosamine, FRAP, plasma creatinine, blood urea nitrogen (BUN), urinary kidney injury molecule-1 (uKIM-1), plasma kidney injury molecule-1 (pKIM-1), albumin/creatinine ratio of urine (ACR), and albuminuria in 5/6 nephrectomised (CKD model) and sham rats.

	Plasma creatinine (*μ*mol/l)	BUN (mmol/l)	pKIM-1 (pg/ml)	uKIM-1 (pg/ml)	ACR (g/mol)	Albuminuria (mg/day)
*Plasma markers of OS*						
TBARS (*μ*mol/g)	-0.42 n.s.	-0.38 n.s.	0.06 n.s.	-0.30 n.s.	0.33 n.s.	0.39 n.s.
AOPP (*μ*mol/g)	-0.12 n.s.	0.05 n.s	-0.15 n.s.	-0.05 n.s.	-0.07 n.s.	-0.08 n.s.
AGEs (g/g)	-0.60^∗^	-0.38 n.s.	-0.38 n.s.	-0.62^∗^	-0.32 n.s	-0.11 n.s.
Fructosamine (mmol/g)	0.10 n.s.	0.17 n.s.	-0.43 n.s.	-0.05 n.s.	-0.06 n.s.	0.07 n.s.
TAC (*μ*mol/g)	0.01 n.s.	0.10 n.s.	0.28 n.s.	0.17 n.s.	0.0 n.s.	-0.08 n.s.
FRAP (*μ*mol/g)	0.07 n.s.	0.10 n.s.	0.06 n.s.	0.12 n.s.	0.14 n.s.	0.12 n.s.
*Urinary markers of OS*						
TBARS (*μ*mol/mmol)	0.23 n.s.	0.44 n.s.	-0.06 n.s.	0.26 n.s.	0.30 n.s.	0.01 n.s.
AOPP (*μ*mol/mmol∗g)	0.23 n.s.	0.06 n.s.	0.38 n.s.	0.70^∗^	0.51 n.s.	-0.10 n.s.
Fructosamine (mmol/mmol)	-0.31 n.s.	-0.32 n.s.	0.13 n.s.	0.17 n.s.	0.72^∗^	0.62^∗^
FRAP (*μ*mol/mmol)	-0.33 n.s.	-0.28 n.s.	0.49 n.s.	0.32 n.s.	0.82^∗∗^	0.69^∗^

^∗^ *p* < 0.05, ^∗∗^*p* < 0.01.

## Data Availability

The Excel or GraphPad data used to support the findings of this study are available from the corresponding author upon request.
